# Look‐up table correction for beam hardening‐induced signal of clinical dark‐field chest radiographs

**DOI:** 10.1002/mp.70307

**Published:** 2026-01-31

**Authors:** Maximilian E. Lochschmidt, Theresa Urban, Lennard Kaster, Rafael C. Schick, Thomas Koehler, Daniela Pfeiffer, Franz Pfeiffer

**Affiliations:** ^1^ Chair of Biomedical Physics, Department of Physics, TUM School of Natural Sciences Technical University of Munich Garching Germany; ^2^ Munich Institute of Biomedical Engineering Technical University of Munich Garching Germany; ^3^ Institute for Diagnostic and Interventional Radiology, School of Medicine and Health, TUM Klinikum Technical University of Munich (TUM) München Germany; ^4^ TUM Institute for Advanced Study Technical University of Munich Garching Germany; ^5^ Philips Innovative Technologies Hamburg Germany

**Keywords:** chest radiography, dark‐field imaging, grating based imaging, medical imaging, medical physics, x‐ray imaging

## Abstract

**Background:**

The microstructure of material at a μm length scale leads to ultra‐small‐angle scattering of X‐rays, which typically occurs, e.g., for lung tissue or some plastic foams. When using an interferometer, this effect alters the visibility of the fringe pattern, which can be detected and resolved by the detector. Thus, the ultra‐small‐angle scattering can be represented as a dark‐field image. For a polychromatic source, the hardening of the source spectrum changes visibility as well, generating an additional fake dark‐field signal by the attenuation of the material on top of the real ultra‐small‐angle scatter‐related dark‐field signal. Consequently, even homogeneous materials without microstructure typically exhibit a change in visibility.

**Purpose:**

The objective of this study is to develop a fast, simple, and robust method to correct dark‐field signals and bony structures present due to beam hardening on dark‐field chest radiographs of study participants.

**Methods:**

The method is based on calibration measurements and image processing. BH by bones and soft tissue is modeled by aluminum and water, respectively, which have no microstructure and thus only generate an artificial dark‐field signal. Look‐up tables were then created for both. By using a weighted mean of these, forming a single LUT, and using the attenuation images, the artificial dark‐field signal and thus the bone structures present are reduced for study participants.

**Results:**

It was found that applying a correction using a weighted LUT leads to a significant reduction of bone structures in the dark‐field image. The weighting of the aluminum component has a substantial impact on the degree to which bone structures remain visible in the dark‐field image. Furthermore, a large negative bias in the dark‐field image–dependent on the aluminum weighting–was successfully corrected.

**Conclusions:**

BH‐induced signal in the dark‐field images was successfully reduced using the method described. The choice of aluminum weighting to suppress rib structures, as well as the selection of bias correction, should be evaluated based on the specific clinical question.

## 1 BACKGROUND

When an X‐ray beam with an energy used in medical imaging passes through matter, several physical interactions can change the beam's properties: Variations in the speed of electromagnetic radiation within the material cause a change in the beam direction, and photoelectric absorption attenuates the beam. Compton scattering is typically also considered a beam‐attenuating effect. In contrast, ultra‐small‐angle x‐ray scattering (USAXS), where the typical scatter angles are so small that scattered photons cannot be distinguished with conventional detectors from non‐scattered photons, is most often considered separately. The scattering power of USAXS is influenced by the size and shape of the material's microstructure.^1^, ^2^, ^3^, ^4^, ^5^, ^6^, ^7^ Phase shift and USAXS cannot be directly resolved by detectors with a typical pixel size of 150μm. However, if precisely arranged gratings are placed in the beam path, all three phenomena can be determined.^8^, ^9^, ^10^

It is important to note that the term “dark‐field signal” or “dark‐field image” typically encompasses all effects that change the visibility, such as the USAXS, beam hardening (BH), and Compton scattering. The objective is to ensure that the dark‐field image exclusively conveys the USAXS information.

Although USAXS and attenuation are two distinct and independent phenomena, in the case of a polychromatic X‐ray beam, attenuation can influence the USAXS channel by hardening the beam on attenuating materials.^11^, ^12^ In the past, methods have been developed to correct the BH‐ induced signal in phase‐contrast and dark‐field imaging using simulations and models. If an object consisted only of soft tissue, the problem could be solved by simulations or a calibration measurement using a soft‐tissue‐like material.^13^ However, this does not work for the two‐material case at hand. Another approach is to define a single calibration curve for multiple materials by weighting the measured transmission with the calculated transmission at the design energy of the imaging system.^12^ However, given the dependence of these weighting factors on the material, the correct weighting factor for material combinations or mixtures remains unknown. Consequently, the applicability of this method to clinical radiographic lung images is also precluded. One further simple method is to apply stronger filtering to the source spectrum $I_{0}\left(E\right)$.^14^ However, this can only slightly mitigate the effect of BH and reduce visibility.

In contrast, this study presents a fast and simple method using a look‐up table (LUT) that enables correction for material combinations of bone and soft tissue in the human thorax. The underlying mathematical principles are illustrated in the following section.

### 1.1 Grating‐based X‐ray interferometry (GBI)

GBI employs the Talbot effect to discern USAXS,^15^ attenuation, and phase^16^ information using coherent X‐ray sources. In this configuration, a phase grating $G_{1}$ with a period of $p_{1}$ generates a Talbot carpet, which exhibits an intensity repetition of the grating period along the beam direction for distances $d_{\text{Talbot}}\left(n\right)=\frac{2p_{1}^{2}}{\lambda }\cdot \left(2n+1\right)\cdot k$ and a parallel beam geometry.^17^ For a cone beam geometry, the distances are adapted to $d_{\text{Talbot}}^{*}=\frac{l\cdot d_{\text{Talbot}}}{l-d_{\text{Talbot}}}$.^18^ In this context, λ represents the wavelength of the monochromatic X‐ray beam, $n\in N_{0}$, and l represents the distance between the focal spot of the coherent X‐ray source and $G_{1}$. The parameter k takes a value of $\frac{1}{4}$ for a $\frac{\pi }{2}$ phase grating and a value of $\frac{1}{16}$ for a π phase grating^19^ generating the Talbot carpet.^20^, ^21^ Nevertheless, alternatively, an attenuation grating may also be employed as $G_{1}$.^22^, ^23^ If a further analyzer grating, designated as $G_{2}$, is now placed at a position $d_{\text{Talbot}}\left(n\right)$, an intensity stepping function can be resolved at the detector for every single pixel. This is achieved by stepping $G_{2}$ perpendicular to the beam direction and to the grating lamellae of $G_{1}$ and $G_{2}$. This function is represented by a first‐order Fourier series, which represents an intensity function depending on the $G_{2}$ stepping position, and the fit parameters are the amplitude, mean value, and phase.^15^ Once the fit parameters for amplitude, mean value, and phase for both the sample and reference scans have been determined for an x‐ray energy E, the attenuation A(E), the dark‐field signal D(E), and the differential phase can be derived from these parameters.^16^, ^24^ The phase information is not further discussed in this work. The mathematical representations of A(E) and D(E) useful for this work are expressed by Equations (1) and (2). In this context, the variables μ and ε represent the linear attenuation coefficient and the linear diffusion coefficient, respectively.^25^ The unit vector pointing from the source to the detector pixel is represented by ${\hat{e}}_{z}$. The constant $c_{D}$ depends on the gratings and the geometry of the setup. V(E) is the visibility of the sample scan, $V_{0}\left(E\right)$ is the visibility of the reference scan, $I_{0}\left(E\right)=\psi_{\text{source}}\left(E\right)\cdot R\left(E\right)$ is the effective source intensity where $\psi_{\text{source}}\left(E\right)$ is the intensity from the X‐ray tube and R(E) is the detector response function. $T\left(E\right)=\exp\left[-\int_{0}^{z_{0}}\mu \left(z{\hat{e}}_{z},E\right)dz\right]$ is the damping and $I\left(E\right)=I_{0}\left(E\right)\cdot T\left(E\right)$ is the intensity detected after passing through a sample or patient.^3^, ^25^, ^26^

(1) $$\begin{array}{cc} & A\left(E\right)=-\ln\left(T(E)\right)=-\ln\left(\frac{I(E)}{I_{0}\left(E\right)}\right)=\int_{0}^{z_{0}}\mu \left(z{\hat{e}}_{z},E\right)dz \end{array}$$

(2) $$\begin{array}{cc} & D\left(E\right)=-\ln\left(\frac{V(E)}{V_{0}\left(E\right)}\right)=-c_{D}\left(E\right)\int_{0}^{z_{0}}\varepsilon \left(z{\hat{e}}_{z},E\right)dz \end{array}$$

GBI can also be done using polychromatic non‐coherent radiation sources by installing an additional attenuation grating, designated as $G_{0}$, behind the source and in front of $G_{1}$. This allows for the beam to be modeled coherently as a periodic array of slit sources and enables constructions for clinical use.^16^

### 1.2 Polychromatic effects

For a polychromatic X‐ray source, visibility $V_{p}$, dark‐field signal $D_{p}$, and attenuation $A_{p}$ are mathematically represented by Equations (3), (4), and (5).^11^ Whereas $V_{\text{0,p}}$ represents the polychromatic visibility of a reference scan at the effective source spectrum $I_{0}\left(E\right)$, and Equation (4) mathematically represents an expression for the damped source‐spectrum‐weighted mean of V(E).^27^

(3) $$\begin{array}{cc} & A_{p}=-\ln\left(\frac{\int_{0}^{\infty }T\left(E\right)\cdot I_{0}\left(E\right)dE}{\int_{0}^{\infty }I_{0}\left(E\right)dE}\right) \end{array}$$

(4) $$\begin{array}{cc} & V_{p}=\frac{\int_{0}^{\infty }V\left(E\right)\cdot T\left(E\right)\cdot I_{0}\left(E\right)dE}{\int_{0}^{\infty }T\left(E\right)\cdot I_{0}\left(E\right)dE} \end{array}$$

(5) $$\begin{array}{cc} & D_{p}=-\ln\left(\frac{V_{p}}{V_{\text{0,p}}}\right) \end{array}$$

In general, we are interested in the USAXS as additional information for the analysis of matter about the microstructure or, in particular, lung diagnostics because this provides information about the microstructure of the sample.^1^, ^2^, ^3^, ^4^, ^5^, ^6^, ^7^, ^28^ Looking at Equation (3), it can be seen that if attenuation decreases monotonically as a function of energy, the mean energy of the detected spectrum I(E) is monotonically raised while passing through the material, which is known as BH. If we then have a further look at Equation (4), it is clear that the dark‐field signal is influenced by this effect as well, as the hardening of I(E) changes the visibility $V_{p}$. Thus, even for a homogeneous material without any microstructure, there is still a change in visibility and, therefore, a dark‐field signal.^11^, ^12^, ^13^, ^14^

## 2 METHODS

### 2.1 Imaging setup

**FIGURE 1:**
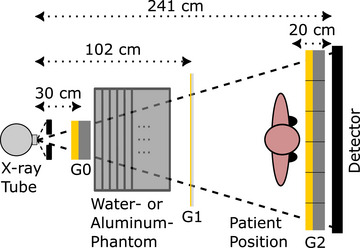
The schematic structure of the fringe‐scanning system is depicted here from an overhead perspective. The interferometer arm is thus positioned within the drawing plane. The collimated X‐ray beam traverses all three gratings. $G_{0}$ serves to render the polychromatic and incoherent X‐ray spectrum coherent. The phase grating $G_{1}$ generates a Talbot carpet. The stepping function is generated by the interferometer arm movement and a slight mismatch of the grating period of $G_{2}$ and the period of the intensity pattern of the Talbot carpet at the position of $G_{2}$, which leads to a moiré pattern at the detector (Table 1). The thickness of the water phantom or the aluminum phantom was incrementally augmented and positioned between $G_{0}$ and $G_{1}$.

The schematic structure of the scanner is illustrated in Figure 1 that uses a fringe‐scanning scheme.^29^, ^30^ Principally, this is a classical radiographic system except for the movable interferometer arm placed within the beam path on which three gratings $G_{0}$, $G_{1}$, and the analyzer grating $G_{2}$ are mounted. While $G_{0}$ and $G_{1}$ are made from single pieces, $G_{2}$ is stitched together in six pieces measuring 6.5cm × 7cm.

Further information about the gratings in terms of fabrication, type, positions, and periods can be seen in Table 1. Here, a slight deviation in the grating period of $G_{2}$ is artificially enforced, which results in a moiré‐fringe pattern at the detector. The stepping function itself is then realized by the upward movement of the interferometer arm during the measurement, as the pattern moves over the detector, which is fixed relative to the interferometer. A total of 195 images are generated during one acquisition of approximately 7s, which are used to determine the stepping function. From that point on, the preliminary dark‐field signal and the attenuation channel can be derived as described in Section 1.1 previously.

**TABLE 1:** Gratings information of the interferometer for the components $G_{0}$, $G_{1}$, and $G_{2}$. The six tiles of $G_{2}$ were aligned using a one‐dimensional stitching method^34^ combined with multiple measurements to ensure sufficient visibility.

	**G** 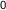	**G** 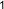	**G** 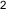
Grating type/influence	Attenuation	Phase‐shift	Attenuation
Number of tiles	1	1	6
Distance from source (cm)	30	102	241
Substrate material	Graphite	Glass	Graphite
Grating material	Gold	Gold	Gold
Substrate height (μm)	1000	200	1000
Gold hight (μm)	250	9.2	250
Grating period (μm)	7.7	10.1	14.8

The polychromatic collimated beam is generated by an X‐ray tube (MRC 200 0508 ROT‐GS 1003, Philips Medical Systems) with a permanent filtration of 2.5mm/75kV Al‐equivalent (IEC 60522/1999), a tube voltage of 70kV, a pulse rate of 30Hz, a pulse length of 17.1ms,^31^ and the large focal spot of 0.8 (IEC 60336) corresponding to a size of 1.2mm × 1.6mm (full width at 15% of maximum).^32^

In addition to the tube's intrinsic filtering properties, other components of the setup provide supplementary filtering before reaching the patient plane. These components are the collimator aperture (R 302 MLP/A DHHS, Ralco) located in front of $G_{0}$ with 1mm/75kV Al‐equivalent filtration (IEC 60522/1999), the dose‐measuring chamber (DIAMENTOR E2, Type 11033, PTW) used to determine the dose‐area product (DAP) behind $G_{0}$ with 0.2mm/75kV Al‐equivalent filtration (IEC 60522/1999), as well as the filtration of the grids $G_{0}$ and $G_{1}$ having a combined Al‐equivalent dose filtration of 7mm Al. To determine the equivalent dose for the two gratings, the dose at the patient position was measured for several Al thicknesses, with both $G_{0}$ and $G_{1}$ removed from the beam path. Based on these measurements, a linear dose‐equivalence curve was established. Subsequently, for an additional measurement with only the $G_{0}$ and $G_{1}$ gratings in the beam path, the corresponding Al‐equivalent dose filtration was derived from this curve. The total filtration before the patient, therefore, is 10.7mm Al equivalence. Subsequently, the beam is subject to further filtration by $G_{2}$, which has an equivalent of 6mm Al. This occurs after the patient and before the detector. The values for the Al‐equivalent dose filtrations of the grids were measured during the initial acceptance inspection of the system and were therefore taken from the acceptance report.

The detector (PIXIUM 4343 F4, Trixell), having a scintillator layer thickness of 600μm of CsI, operates with a pixel‐binning of 3×3 resulting in a physical pixel size of 444μm. For study participants who are always placed at a contact plane with a distance of 20cm to the detector, this results in an effective pixel size of about 400μm in a posterior‐anterior (p.‐a.) position. This can vary slightly depending on the size of the patient and the distance to the detector. For a reference patient who is scanned in a p.‐a. position, the effective dose is 35μSv.^33^

### 2.2 Correction of BH‐induced dark‐field signal

When considering the X‐ray attenuation by a human thorax, it is appropriate to classify all components whose X‐ray attenuation is non‐negligible into two main types based on their attenuation characteristics: bone and soft tissue, which do not or only to a very small extent cause real USAXS. Bone and soft tissue both strongly contribute to the hardening of the source spectrum $I_{0}\left(E\right)$, resulting in a BH‐induced dark‐field signal on top of the real USAXS. However, this component of the dark‐field signal does not convey any information about tissue microstructure and should therefore be removed from the dark‐field image. Lung tissue, while part of soft tissue, also contributes to the dark‐field signal through real USAXS at the alveolar structures. This USAXS‐based signal provides valuable insights into the microstructure and represents the component we aim to isolate and visualize in the final dark‐field image.

Considering Equation (5), it can be seen that mathematically, the fraction can be expanded by the BH‐induced visibility change caused by an attenuator, so that the expression can be decomposed into a USAXS term $D_{p}^{'}$ and a BH‐induced term $D_{p}^{\text{BH}}$ (Equation 6).

(6) $$\begin{array}{c} D_{p}=-\ln\left(\frac{V_{p}}{V_{\text{0,p}}^{\text{BH}}}\cdot \frac{V_{\text{0,p}}^{\text{BH}}}{V_{\text{0,p}}}\right)=-\ln\left(\frac{V_{p}}{V_{\text{0,p}}^{\text{BH}}}\right)-\ln\left(\frac{V_{\text{0,p}}^{\text{BH}}}{V_{\text{0,p}}}\right)=D_{p}^{'}+D_{p}^{\text{BH}} \end{array}$$

Here we introduced $V_{\text{0,p}}^{\text{BH}}$ as the reference fringe visibility measured using the same hardened X‐ray spectrum as with the sample. Using this reference, we can eliminate the artificial dark‐field signal caused by the energy‐dependent fringe visibility of the interferometer. The main goal now is to estimate $D_{p}^{\text{BH}}$ from the actual measurement and some calibration measurements.

Here we pursue the concept to use a single LUT to estimate $D_{p}^{\text{BH}}$. The concept makes use of the fact that the beam hardens monotonically with increasing attenuation. Thus, the measured attenuation $A_{p}$ can be used as a surrogate for the beam‐hardness, i.e., as the entry variable of the LUT. For a pure attenuator without microstructure, Equation (6) simplifies to $D_{p}=D_{p}^{\text{BH}}$. This allows the BH‐induced signal $D_{p}^{\text{BH}}$ and the corresponding attenuation $A_{p}$ to be directly measured for various attenuator thicknesses. To calculate the LUTs for the known base materials without any microstructure, they are subjected to individual measurements for different layer thicknesses placed right after $G_{0}$ and before $G_{1}$ (Figure 1). The reason for the different positioning compared to the patient position is that this way, the entire field of view (FOV) can be covered with minimal material, and the exchange of individual layers is easier. Due to very similar attenuation properties, it is common practice in computed tomography (CT) to model soft tissue‐induced BH by water^35^ and bone by aluminum.^36^, ^37^ Therefore, these two materials were used to determine the LUTs for both. In this context, the calibration measurements entail the acquisition of an attenuation image, designated as $A_{p}\left(u,v\right)$, and a dark‐field image $D_{p}\left(u,v\right)$ for each material and each layer thickness, under the standard measurement protocol. However, given that the measured base materials lack any microstructure and thus do not induce USAXS, any measured dark‐field signal is entirely attributable to the hardening of the source spectrum, $I_{0}\left(E\right)$.

From the obtained data points, a LUT for pure water 
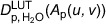
 and for pure aluminum $D_{\text{p, Al}}^{\text{LUT}}\left(A_{p}\left(u,v\right)\right)$ are created for each pixel (u,v) using spline fits, which also represents a metric to quantify the BH effect and how different the response is between bone (Al) and soft tissue (water). These were then combined by a weighted mean to a single LUT $D_{\text{p,}\;\omega_{\text{Al}}}^{\text{LUT}}\left(A_{p}\left(u,v\right)\right)$ where $\omega_{\text{Al}}\in \left[0,1\right]$ represents the contribution of aluminum (Equation 7). The reason for choosing to perform this correction pixel by pixel is that the intensity spectrum $I_{0}\left(E\right)$ is not globally constant due to the heel effect of the X‐ray tube, and the LUT is expected to vary pixel by pixel.

(7)

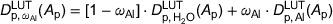

The final correction procedure for the measurements of the study participants is described by Equation (8). Here, the BH‐induced dark‐field signal, determined using the LUT and the attenuation image $A_{p}\left(u,v\right)$, is calculated and subtracted from the measured dark‐field image $D_{p}\left(u,v\right)$. The term $D_{\text{BHC-bias}}$ is a correction for a bias or overcorrection in regions of pure water or soft tissue that might be present if a weighted single LUT with $\omega_{\text{Al}}>0$ is chosen. Since for X‐ray chest radiographs, the exact regions of pure water or soft tissue are unknown and the attenuation changes in the image, this term needs to be chosen for regions of pure water or soft tissue, but is added for all pixels (u,v). Fundamentally, the term $D_{\text{BHC-bias}}$ corresponds to a re‐windowing of the image; however, in contrast to conventional windowing, the correction here can be mathematically justified since, in principle, $D_{\text{BHC-bias}}$ can be calculated for a region of pure water by the difference between the LUT of pure water and the weighted single LUT used (Equation 9).

(8) $$\begin{array}{cc} & D_{p}^{'}\left(u,v\right)=D_{p}\left(u,v\right)-D_{\text{p,}\;\omega_{\text{Al}}}^{\text{LUT}}\left(A_{p}\left(u,v\right)\right)+D_{\text{BHC-bias}}\;\forall \left(u,v\right) \end{array}$$

(9)

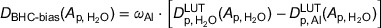

Thus, the goal of this work is to find optimal value pairs of $\omega_{\text{Al}}$ and $D_{\text{BHC-bias}}$ to achieve the best reduction of the BH‐induced dark‐field signal and the bony structures in the final images. Therefore, study participant measurements were processed with different settings of this beam hardening correction (BHC) method and analyzed. Please note that the attenuation signal is used for BHC of the dark‐field images, but the attenuation image itself was not BH corrected, as this is not a common practice in conventional radiography.

### 2.3 Phantom constructions and phantom measurement

For the base materials, total thicknesses of 24.0cm for water and 6.0cm for aluminum were chosen. For the aluminum phantom, several aluminum sheets with thicknesses of 0.2cm and 0.1cm were used. In total, 13 different thicknesses were realized.

**FIGURE 2:**
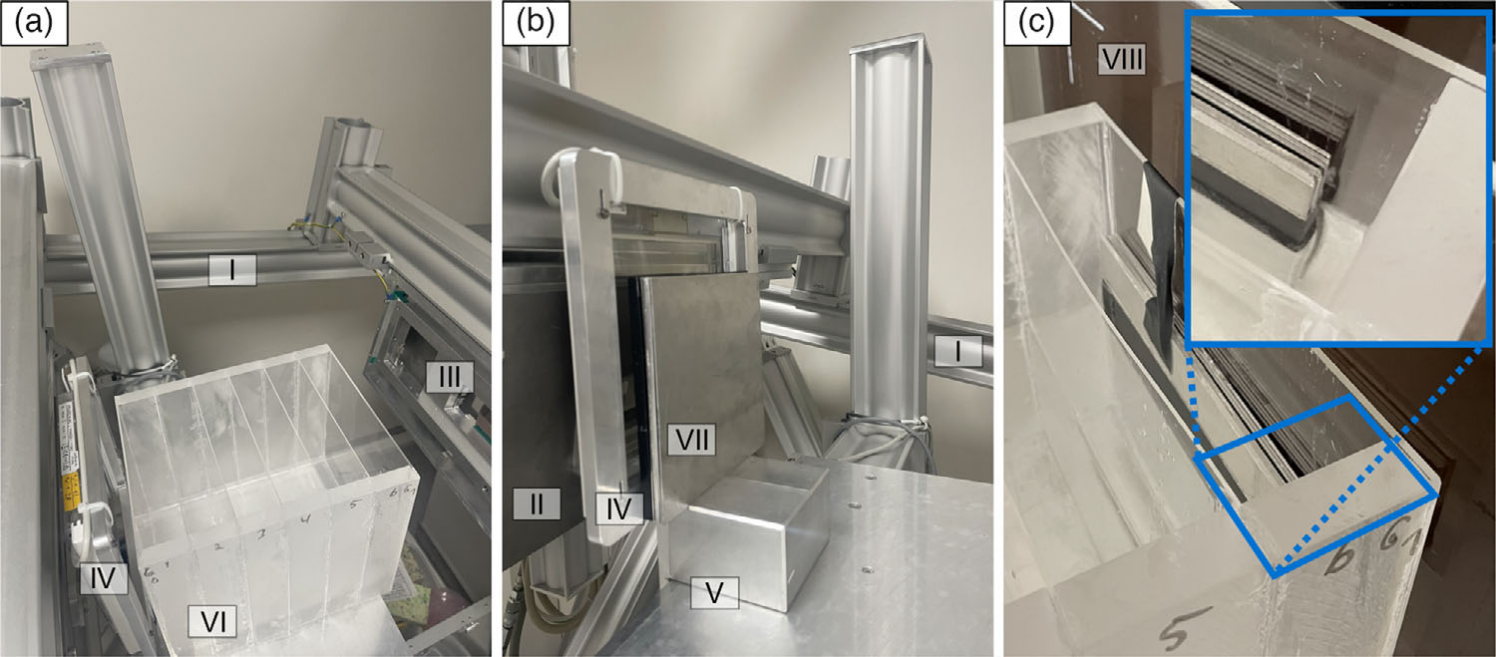
(a) Illustrates the water tank for the soft tissue (behind $G_{0}$ and before $G_{1}$) for the calibration. (b) Illustrates the aluminum plates for the same purpose and position. (c) Shows an 7mm aluminum edge in the water phantom with two chambers filled, which is used to validate the method. Figure legend: **I**. Interferometer arm, **II**. $G_{0}$ with lead shield, **III**. $G_{1}$ with lead shield, **IV**. Dose‐measuring chamber, **V**. Sample table with platform, **VI**. Water phantom, **VII**. Aluminum phantom, **VIII**. Patient position.

In the case of the water phantom, a corresponding water container was made of Poly(methyl 2‐methylpropenoate) (PMMA), which has six separate chambers, each with a thickness of 4cm. This means that the chambers can be gradually filled with water from measurement to measurement, allowing measurements to be made for a total of seven different water thicknesses. The wall thickness of the PMMA was 0.1cm for the inner partitions and 0.15cm for the end walls. All individual material thicknesses of aluminum and water are summarized in Table 2. Photos of the phantoms are shown in Figure 2a,b. Before applying and demonstrating the correction method on study participants, the new correction was validated using an aluminum edge with a thickness of 7mm, which was inserted into a water phantom filled with two chambers (8cm). For this validation, the entire phantom was positioned at the patient position (Figure 2c).

**TABLE 2:** Material thicknesses of the water‐ and aluminum‐phantoms.

Thickness number	1	2	3	4	5	6	7	8	9	10	11	12	13
Water (cm)	0 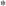	0	4	8	12	16	20	24	—	—	—	—	—
Aluminum (cm)	0.0	0.2	0.4	0.6	0.7	1.1	1.4	2.1	2.5	3.0	3.9	5.0	6.0

* Empty image (measured without the water container)

### 2.4 Application on study participants

In order to ascertain the optimal aluminum weighting $\omega_{\text{Al}}$ of the single LUT (7) and the level of the BHC‐bias correction $D_{\text{BHC-bias}}$, three dark‐field radiographs in p.‐a. orientation were corrected by the BHC method presented in 2.2 for different combinations of $\omega_{\text{Al}}$ and $D_{\text{BHC-bias}}$. The goal is to find the best reduction of the bony structures and BH‐induced dark‐field signal ‐ the best improvement of image quality. For this purpose, analyses were conducted on both the lung regions and the extrapulmonary areas near the clavicle, where no real USAXS signal is anticipated due to the lack of microstructural features.

The larger prospective study out of which the three study participants for this work were taken was conducted in accordance with the Declaration of Helsinki (as revised in 2013). Approval of the institutional review board and the national radiation protection agency was obtained prior to this study (Ethics Commission of the Medical Faculty, Technical University of Munich, Germany; reference no. 587/16S). Participants gave their written informed consent.

To ensure the target detector dose of 3.75μGy was achieved, the required tube current for each study participant was individually determined based on a calibration curve and dose reference data obtained from the conventional imaging system.^38^

Care was taken to include one participant with a heavy physique and two with medium or light physiques in order to cover the typical range of X‐ray attenuation observed in the human body. These participants were also chosen to include one healthy participant and two participants with chronic obstructive lung disease (COPD) to ensure that the typical range of dark‐field signal in humans was also represented. The gender, height, and weight are given in Table 3. For participants with COPD, it could be shown previously that the fringe scanning prototype provided dark‐field images that improved the diagnostics.^39^, ^40^, ^41^

**TABLE 3:** Gender, weight, and height of one healthy and two COPD study participants.

	Healthy	COPD 1	COPD 2
Gender	Male	Male	Male
Weight (kg)	96.2	64.1	64.3
Height (cm)	170	173	175

### 2.5 Additional image pre‐corrections

For study participants, additional standard corrections were implemented for all measurements conducted on the setup before the BHC presented here was applied. Firstly, Compton scatter caused by the gratings in the beam path, which is dominated by $G_{2}$, are considered. This correction is based on a scatter kernel, which was defined using simulations using the Geant4 toolkit (version 10.06.p03).^31^, ^42^ Compton scattering caused by study participants is corrected by SkyFlow (Philips Medical Systems,^43^) adapted to the scanning system. Finally, the detector itself has a non‐negligible detector crosstalk. A specific kernel was also defined for this purpose, which removes this crosstalk.^31^ Moreover, motion corrections for the heart, vibration corrections resulting from interferometer arm movement, and bias correction are applied to the images.^24^, ^44^ Images corresponding to the individual corrections can be found in the respective references.

The Compton scatter correction using SkyFlow had to be manually turned off for the water and aluminum phantoms due to the changed positioning compared to the study participants. Instead, the sample scatter for each layer thickness was simulated with the Geant4 toolkit. The reason for tuning off SkyFlow is that the software is only adapted for positioning objects at the patient position 20cm before the detector. For varying distances, the scatter must be manually simulated. As was the case for the study participants, all other corrections remained activated.

It is important to note that, prior to the BHC described in this work, all other corrections mentioned here had already been applied. Therefore, effects such as Compton scatter do not influence this correction.

## 3 RESULTS

### 3.1 LUTs and phantom measurement

**FIGURE 3:**
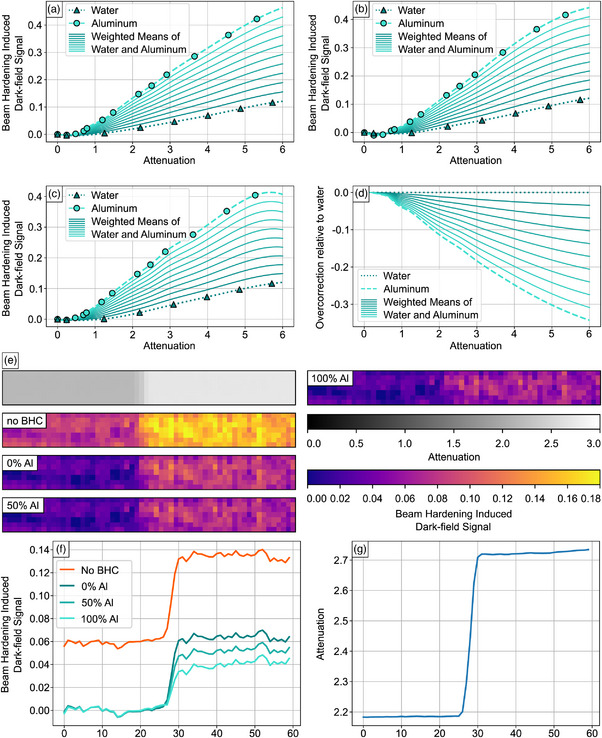
(a) Illustration of the dark‐field signal induced by BH for the aluminum and water container phantoms (Table 2), plotted over the corresponding attenuation for the middle, (b) for the upper and (c) for the bottom region of the detector. The LUTs for aluminum and water, generated by a spline‐function fit, and different weighted averages of both are displayed. (d) Shows the overcorrection compared to pure water regions for different aluminum weightings $\omega_{\text{Al}}$ and is mathematically described by (9).(e) Attenuation and dark‐field images of the Al‐edge phantom shown in Figure 2c without a correction and for LUT weighting values $\omega_{\text{Al}}$ of 0% Al, 50% Al, and 100% Al. (f) and (g) show line plots through the dark‐field and attenuation images shown in E.

As previously described, the spline‐fit functions or LUTs are created pixel by pixel. Here, a representative region of interest (ROI) was selected from the center, top, and bottom regions of the detector to show the spline fits or LUTs for pure water, pure aluminum, and weighted single LUTs in increments of 10% of $\omega_{\text{Al}}$ as described by Equation (7) and shown in Figure 3a–c. For the center region, the BHC‐bias or overcorrection relative to pure water is shown in Figure 3d. All single LUTs and the plotted BHC‐bias serve as the basis for the various corrections to the study participants and the phantom measurement that is shown in Figure 3e–g, where the attenuation signal, the uncorrected dark‐field signal as well as dark‐field signals at several different correction levels $\omega_{\text{Al}}$ of the water edge depicted in Figure 2c are presented as images and line plots.

### 3.2 Application on study participants

**FIGURE 4:**
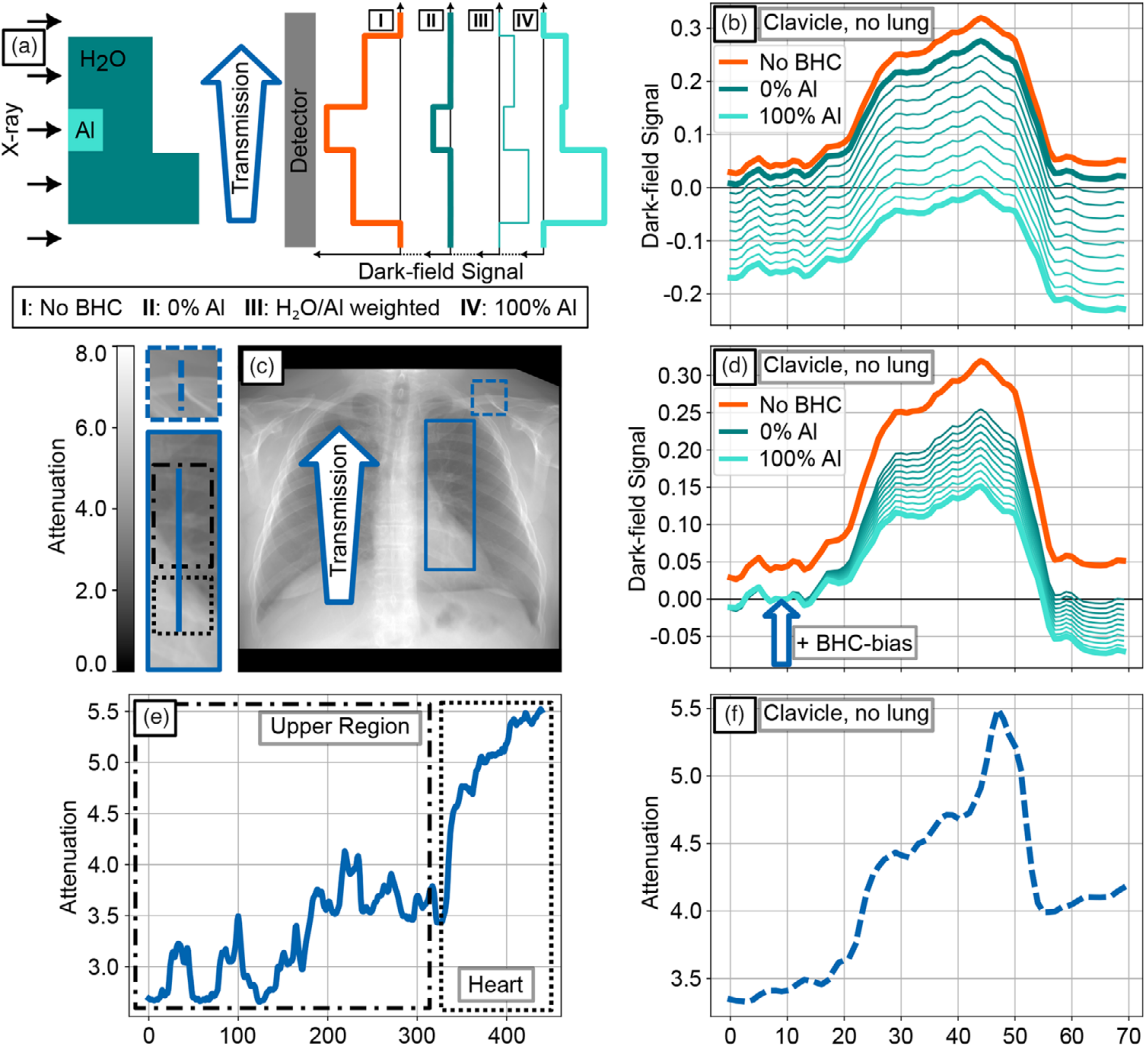
(a) Illustration of the changing bone level to pure water with different weightings of the aluminum and water LUT to a single LUT and different transmission levels. (b) Lineplots of the BH‐induced dark‐field signal and (f) The attenuation signal through the left clavicle for different LUT weightings and for the healthy patient with a heavy physique. (d) represents a correction of the BHC‐bias for the lower attenuation region. (e) Line plot of the range of the attenuation from the upper part of the lung down to the region of the heart. (c) The attenuation image of the healthy patient shows where the line plots were taken.

In an initial analysis, the general impact of this BHC and the selection of the weighting parameter $\omega_{\text{Al}}$ on the occurrence of false USAXS signals was investigated using data from the healthy study participant. A line profile across the clavicle bone, located outside the lung region (Figure 4b), clearly demonstrates that increasing the aluminum weighting $\omega_{\text{Al}}$ leads to a reduction in the bone step, thereby enhancing signal homogeneity in this region.

However, this improvement is accompanied by a systematic increase in BHC‐bias, with the signal being progressively shifted toward negative values as $\omega_{\text{Al}}$ increases. This observation confirms that the magnitude of the BHC‐bias is not solely dependent on the weighting factor but is also influenced by the local attenuation level, as also illustrated in Figure 3d. This dependency is particularly evident in Figure 4d, where the BHC‐bias parameter $D_{\text{BHC-bias}}\left(A_{p}\right)$ was individually adjusted for each aluminum weighting such that the attenuation region to the left of the bone step was corrected to zero, effectively eliminating the overcorrection. Nevertheless, a residual overcorrection remains on the right side of the bone step, corresponding to the differential attenuation between the two regions. This phenomenon is further exemplified in Figure 4a. The clinical relevance of attenuation‐dependent overcorrection becomes even more apparent when examining a line profile extending from the upper lung region to the cardiac region (Figure 4c,e). A gradual increase in attenuation is observed along this path. Consequently, an exact bias correction term $D_{\text{BHC-bias}}$ strongly depends on attenuation for clinical radiographic dark‐field images.

**FIGURE 5:**
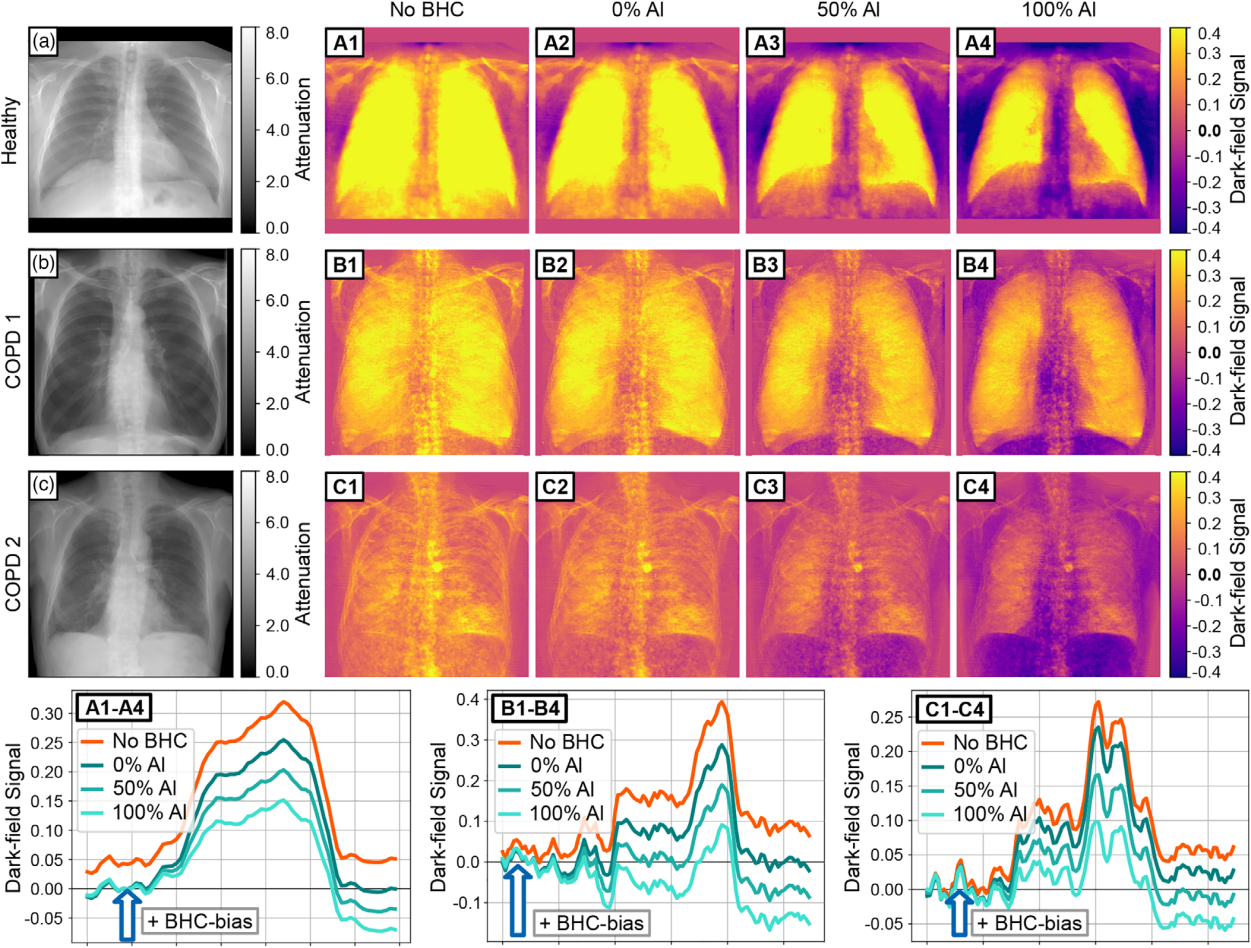
(a–c) Attenuation images of the healthy and the two COPD patients. Next to them, the effect of the single LUT corrections on the images regarding the overcorrection described in more detail in Figure 4 is illustrated for the LUT weightings of **A2,B2,C2**. 0% aluminum, **A3, B3, C3**. 50% aluminum, **A4,B4,C4**. 100% aluminum, and **A1, B1, C1**. without any BHC correction. For all correction stages shown here, line plots were generated for each patient along the clavicle, and the BHC bias was applied accordingly. These plots are labeled **A1–A4**, **B1–B4**, and **C1–C4**.

The effects of aluminum weighting and attenuation on the overall dark‐field image are presented in Figure 5 for all three study participants. As in previous observations, it becomes particularly evident–especially in regions outside the lung–that higher attenuation signals, at constant aluminum weighting, generally lead to an increased BHC‐bias. Conversely, for a given attenuation level, an increase in aluminum weighting also results in a higher BHC‐bias and a reduction of the bone steps.

**FIGURE 6:**
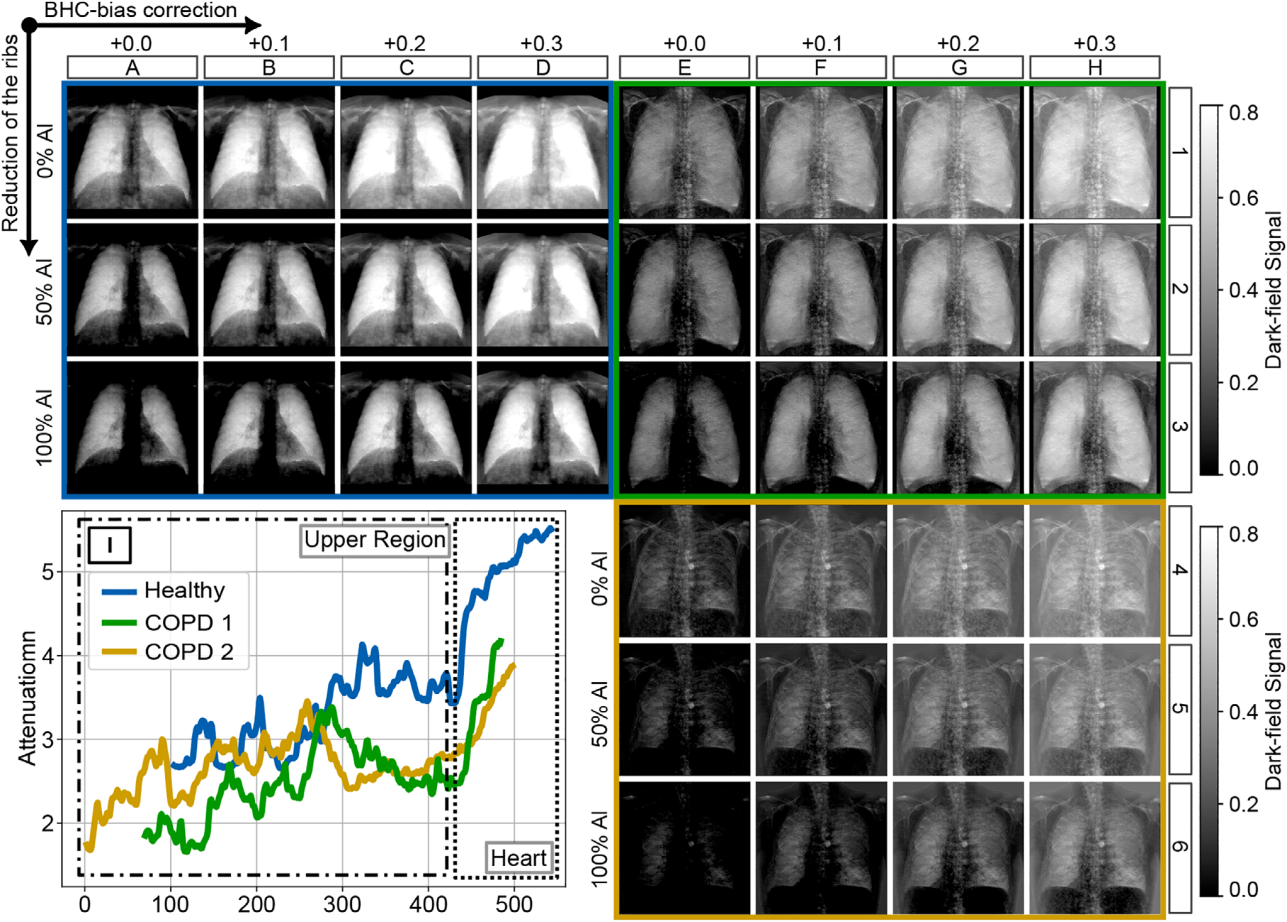
**A1–H6**. Illustrates the healthy patient (blue) and the two COPD patients (green and yellow) being processed for different bias correction levels and for the LUT weightings of 0%, 50%, and 100% of aluminum. **I**. Represents line plots of the attenuation images of all patients from the upper part of the lung to the region of the heart.

Overall, it can be seen that the choice of $\omega_{\text{Al}}$ governs the degree of the bone step reduction, while the BHC‐bias parameter $D_{\text{BHC-bias}}$ compensates for overcorrection within a targeted attenuation range. Both parameters are compared across all three patients in Figure 6, alongside the corresponding attenuation profiles extending from the upper lung region to the cardiac region.

## 4 DISCUSSION

It should be noted that the method presented in this work, as described by Equations (6), (7), (8), and (9), is a pixel‐wise correction of BH–induced dark‐field signals, which needs parameters chosen before, since the material composition of bone and soft tissue is not known for every pixel. However, the method has the ability to significantly reduce both the fake BH signal and bone structures in the dark‐field image for regions with similar attenuation, provided that the parameters $\omega_{\text{Al}}$ and $D_{\text{BHC-bias}}$ are appropriately chosen. This effect is particularly evident in the overview of all three study participants shown in Figure 6. Furthermore, it becomes clear that there is no single pair of values for both parameters that consistently yields the best image correction. For each study participant, the attenuation in the lung region must be assessed in order to determine the appropriate bias correction term $D_{\text{BHC-bias}}$ using the plot shown in Figure 3d, and based on the selected aluminum fraction $\omega_{\text{Al}}$. Depending on the clinical question, these parameters have to be chosen, or compromises in both parameters may be acceptable to achieve a global rather than region‐specific correction of the dark‐field image.

One reason this method was applied on a pixel‐wise basis is the presence of the heel effect. Due to the mounting configuration of the X‐ray tube, the heel effect is expected to occur vertically along the detector in such a way that the mean energy of the source spectrum $I_{0}\left(E\right)$ becomes progressively higher from the upper to the lower part of the detector. This can also be observed in Figure 3a–c as a horizontal shift in the measurement points used to determine the LUTs for pure water and pure aluminum. However, this effect is minor, and therefore only the central region of the detector was considered for the overcorrection plot in Figure 3d and for further usage of the bias correction term $D_{\text{BHC-bias}}$.

As already mentioned in Section 1, several methods have been published in the past. However, all of these approaches have limitations when dealing with material combinations that exhibit different spectral properties and are therefore not suitable for clinical applications. Whereas the method presented in this work represents an improvement for imaging scenarios involving combinations of bone and soft tissue, particularly in clinical radiographic lung imaging. At the same time, it encourages further research in this direction. One improvement might be to use the attenuation image for bone segmentation, resulting in bone‐ and soft tissue‐masks. For each mask, different weightings of the LUTs could then be chosen and applied to the dark‐field image being corrected for the fake dark‐field signal. In this case, as well, a weighting would need to be estimated for the regions identified as containing bone. However, such an approach would eliminate the need for a separate bias correction, since it would be known which regions are affected by soft tissue only and which involve a combination of materials, including bone. A further improvement might be a pixel‐wise, physically exact correction, without any discussion about the LUT weightings, which is only possible if attenuation can be separated for each material. Using spectral radiographic measurements, the artificial dark‐field signal could then be corrected using a model of the dark‐field and attenuation signals, taking into account all materials involved for each pixel.

In principle, one could consider applying this projection‐based BHC to future dark‐field CT projects as well. However, it should be noted that the method presented here might only be applied prior to 3D reconstruction, as it is purely projection‐based. Given the large number of projections involved, this approach may impose an excessive computational effort, suggesting that an alternative method operating on the reconstructed 3D volume will likely be necessary. Nevertheless, commercial CT systems typically perform a scout scan before each measurement to define the relevant FOV. Since this overview scan represents a single 2D projection, the method presented in this work can be applied to scout scans and may help reduce the appearance of bone structures, thereby improving image quality.

## 5 CONCLUSIONS

In conclusion, we proposed a BHC using a single LUT to reduce the artificial BH signal and the bony structures for dark‐field chest imaging to a large extent. The approach is LUT‐based semi‐empirical, as the content of the LUT is based on physical measurements, but the specific weighting to generate the final LUT values is optimized using qualitative image quality assessment. Practically, two parameters and their respective effects on the corrected images must be considered when applying this type of correction. First, the aluminum weighting, which determines the extent to which rib structures are suppressed, and second, the BHC bias correction term compensating for the overcorrection introduced by this method. The appropriate level of BHC bias correction is strongly dependent on the local attenuation and can optionally be done by re‐windowing of the dark‐field images as well. Therefore, for each clinical question, the parameter configuration must be individually assessed to ensure sufficiently high image quality, or compromises in both parameters may be acceptable to achieve a global rather than region‐specific correction of the dark‐field image. In principle, however, an optimal configuration can always be found for each region of the lung, enabling effective correction of the BH‐induced dark‐field signal and bony structures.

## CONFLICT OF INTEREST STATEMENT

Thomas Koehler is an employee of Philips Innovative Technologies. The other authors declare no conflicts of interest.
